# ProInflam: a webserver for the prediction of proinflammatory antigenicity of peptides and proteins

**DOI:** 10.1186/s12967-016-0928-3

**Published:** 2016-06-14

**Authors:** Sudheer Gupta, Midhun K. Madhu, Ashok K. Sharma, Vineet K. Sharma

**Affiliations:** Metagenomics and Systems Biology Group, Department of Biological Sciences, Indian Institute of Science Education and Research Bhopal, Bhopal, Madhya Pradesh India

**Keywords:** Proinflammatory, Antigens, Prediction, Vaccine, Machine-learning

## Abstract

**Background:**

Proinflammatory immune response involves a complex series of molecular events leading to inflammatory reaction at a site, which enables host to combat plurality of infectious agents. It can be initiated by specific stimuli such as viral, bacterial, parasitic or allergenic antigens, or by non-specific stimuli such as LPS. On counter with such antigens, the complex interaction of antigen presenting cells, T cells and inflammatory mediators like IL1α, IL1β, TNFα, IL12, IL18 and IL23 lead to proinflammatory immune response and further clearance of infection. In this study, we have tried to establish a relation between amino acid sequence of antigen and induction of proinflammatory response.

**Results:**

A total of 729 experimentally-validated proinflammatory and 171 non-proinflammatory epitopes were obtained from IEDB database. The A, F, I, L and V amino acids and AF, FA, FF, PF, IV, IN dipeptides were observed as preferred residues in proinflammatory epitopes. Using the compositional and motif-based features of proinflammatory and non-proinflammatory epitopes, we have developed machine learning-based models for prediction of proinflammatory response of peptides. The hybrid of motifs and dipeptide-based features displayed best performance with MCC = 0.58 and an accuracy of 87.6 %.

**Conclusion:**

The amino acid sequence-based features of peptides were used to develop a machine learning-based prediction tool for the prediction of proinflammatory epitopes. This is a unique tool for the computational identification of proinflammatory peptide antigen/candidates and provides leads for experimental validations. The prediction model and tools for epitope mapping and similarity search are provided as a comprehensive web server which is freely available at http://metagenomics.iiserb.ac.in/proinflam/ and http://metabiosys.iiserb.ac.in/proinflam/.

**Electronic supplementary material:**

The online version of this article (doi:10.1186/s12967-016-0928-3) contains supplementary material, which is available to authorized users.

## Background

The role of peptides as therapeutic agents has gained considerable importance recently, and more than 7000 natural peptides have been reported which play a pivotal role in human physiology and have different applications such as, vaccines, and other immunotherapeutics [1]. However, in addition to the desired action, these peptides may show undesirable immuno-activity, for example B cell or T cell activation and other proinflammatory events [2–4]. Similarly in nature, different infectious agents also harbor immunomodulatory properties present in their proteins, which help them in initiation and progression of the disease [5, 6]. Several examples of proinflammatory reactions are known where the pathogens get advantage of inflammation caused by their antigen. A well-known example is the proinflammatory response induced by the peptide Hp(2–20) of *Helicobacter pylori* which induces proinflammatory activities such as, recruiting and activating various immune cells like neutrophils and monocytes, upregulation of integrins (Mac-1) and activation of the oxygen radical producing NADPH-oxidase. This leads to destruction of host mucosal tissue along with reduction in the viability and function of antineoplastic lymphocytes [7]. Similarly, the peptide gG-2p20, which corresponds to amino acids 190–205 of glycoprotein G-2 of Herpes Simplex Virus-2 (HSV-2), induces proinflammatory effects by recruiting and activating the phagocytic cells. This, in turn, leads to reduced function and viability of NK cells [8]. Since NK cells constitute early line of defense and particularly important in protection against HSV-2, such proinflammatory reaction caused by gG-2p20 peptide leads to HSV-2 infection. Furthermore, there are examples of other physiological diseases, such as transmissible spongiform encephalopathies (TSEs), where prion peptide PrP(106–126) increases the pathogenicity due to its proinflammatory nature [9]. Similarly, LL-37, a 37 amino acid proinflammatory peptide generated from hCAP18 protein, has a role in pathogenesis of rheumatoid arthritis, systemic lupus erythematosus, atherosclerosis etc. [10]. Another example of proinflammatory peptide is C-peptide, a cleavage product of proinsulin which is used in peptide-therapeutics. It has a proinflammatory response in different tissues and this property leads to inflammation in kidney and vasculature, worsening the disease in long term [11].

The above evidences of proinflammatory property of peptide sequences underscore the correlation between amino acid sequence and its proinflammatory behavior. To the best of authors knowledge, there are no computational studies reported till date where any sequence-based signature or feature has been investigated which could be responsible for proinflammatory behavior of a peptide. Although, several studies have focused on the prediction of different kind of immune epitopes, such as B cell epitopes [12–14], T cell epitopes [15–17], MHC binders [18], IL4-inducing peptides [19], IFN-gamma inducing MHC binders [20] and allergenicity [21, 22], there is no study known where the sequence-based features have been examined to determine the proinflammatory nature of peptides. In this work, we have analyzed amino acid sequence of experimentally validated proinflammatory epitopes (PiEs) in contrast to non-proinflammatory epitopes (NPiEs) and developed a machine learning-based classification method incorporating the sequence-based features, to predict the proinflammatory nature of peptides and proteins.

## Results and discussion

**Fig. 1:**
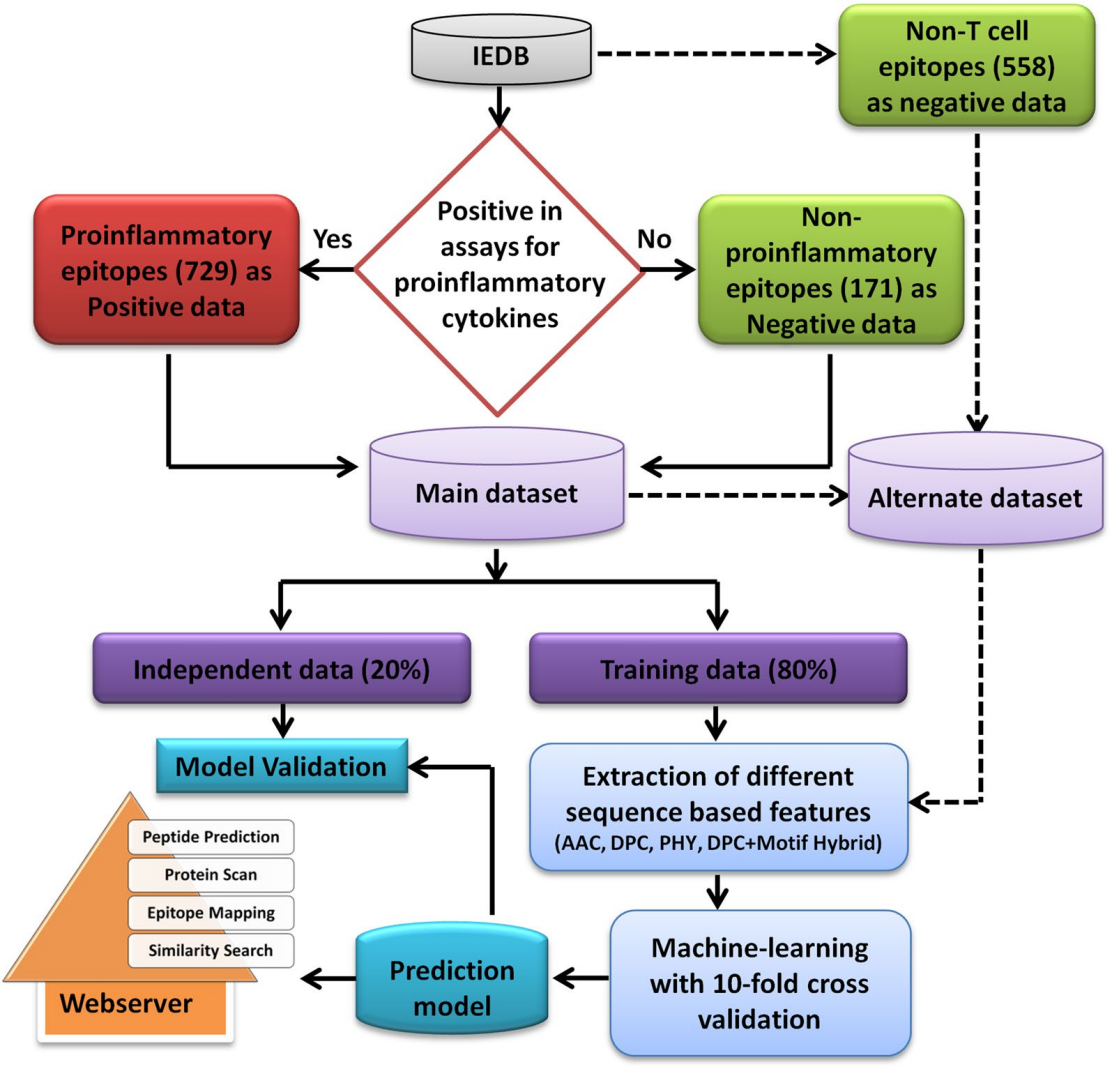
Flowchart showing steps involved in the development of prediction model and web server

The induction of proinflammatory immune response may be a desirable or undesirable property of peptide therapeutics. There are examples of therapeutic peptides where inflammation is a desirable property [3, 23]. However, examples like C-peptide have an undesirable proinflammatory behavior, which worsen the disease [11]. The aim of this study is to develop an in silico method for predicting PiEs. In this study, we have analyzed the sequence-based properties which may contribute to its proinflammatory nature. Although in the past, several studies have been carried out on allergenic proteins/peptides [21, 22], toxic peptides [24], MHC binders [18], CTL epitopes [17], and B cell epitopes [12]; this study focus on investigating the basic property of peptide antigens to initiate proinflammatory cascade, which involves recruiting several immune cells, activation of complement proteins and communication via different immune mediators, which are also known as cytokines. The cytokines, such as IL1α, IL1β, TNFα, IL12, IL18 and IL23, are considered as proinflammatory cytokines [25], which are established mediators measured during a proinflammatory reaction assay. In this study, the experimentally validated epitopes which are assayed positive for these cytokines were considered as PiEs. The epitopes which gave negative assay were considered as NPiEs (Fig. 1). The compositional and motif-based analysis were carried out on the main dataset, however, the prediction models were developed using both main and alternate dataset, as discussed in methods section.

### Compositional analysis

**Fig. 2:**
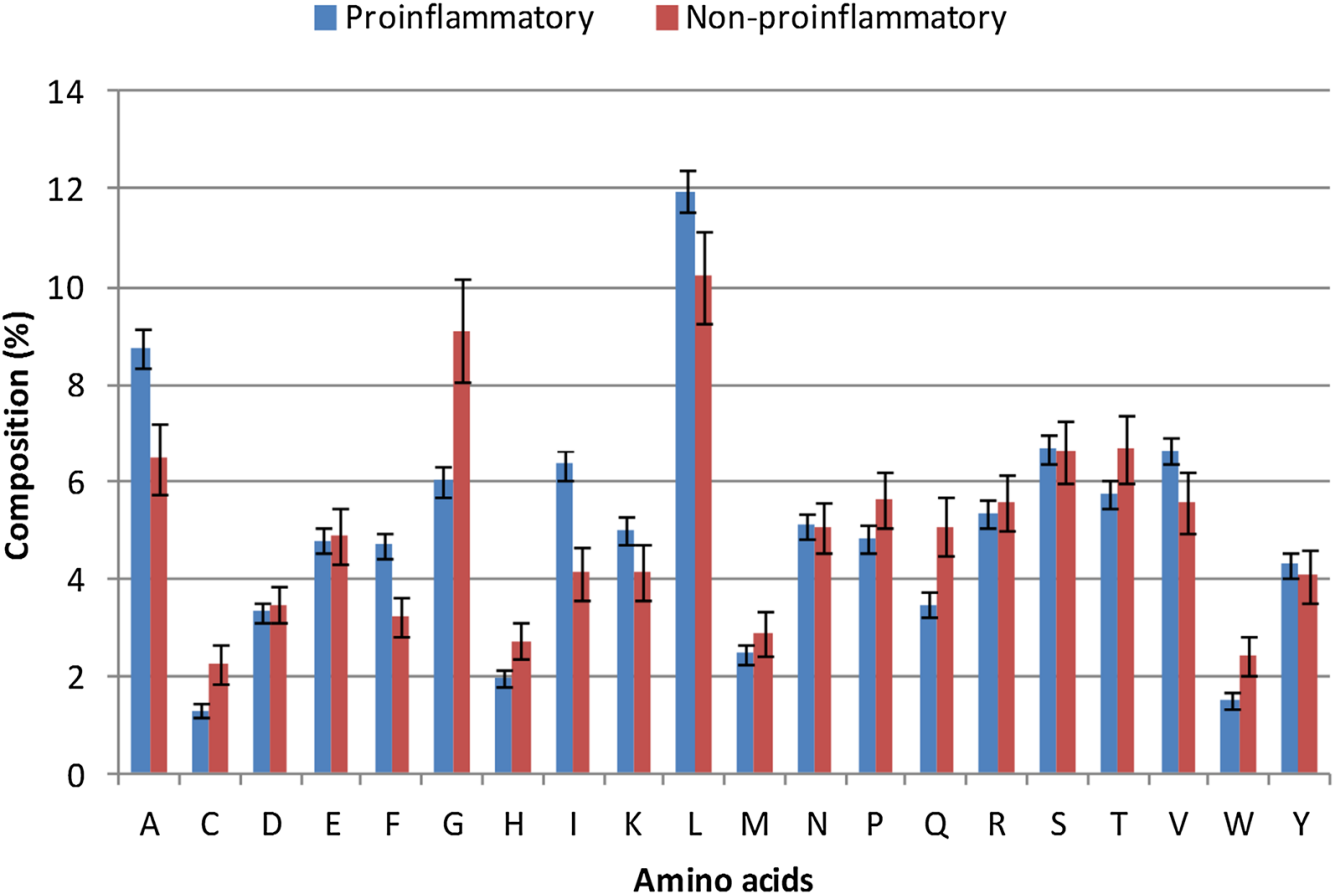
Compositional analysis of proinflammatory and non-proinflammatory epitopes

In order to examine the abundance of amino acids in PiEs as compared to NPiEs, amino acid composition was computed for both the epitope classes. The compositional analysis revealed that the average composition of Ala, Phe, Iso, Leu and Val amino acids is higher in PiEs as compared to NPiEs (Fig. 2; Additional file 1: Table S1), whereas, the amino acids Cys, Gly, Pro and Thr are less abundant in PiEs. It suggests a preferential abundance of some amino acids in PiEs. Similarly, the dipeptide composition was examined in both the classes and several dipeptides were observed to be significantly abundant (Welch’s t test, p < 0.05) in PiEs. The composition of 91 dipeptides were found to differ significantly in PiEs as compared to NPiEs, of which the dipeptides AF, DA, GF, IN, KA, KD, RK, RM, TL and YA are the top ten dipeptides which showed the most significant differences in composition. Of these 91 significantly different dipeptides, AF, FA, FF, PF, IV, IN were also observed to be the most abundant in PiEs (Additional file 2: Table S2).

The composition-based analysis revealed that in PiEs, most of the preferred residues (Ala, Phe, Iso, Leu and Val) are aliphatic or hydrophobic amino acids. Similarly, the significant dipeptides, discussed in the above section, mostly included pairs of aliphatic or hydrophobic amino acids in different local orders.

### Motif analysis

The proinflammatory immune response requires the activation of T cells by presentation of peptide bound to MHC molecule. Several key amino acids of the peptide bind at certain positions in the binding core of MHC which suggests that peptide may contain specific motif required for binding. In order to investigate if there are particular sequential motifs present in PiEs, the motifs were searched in the training datasets using MERCI software as described in the Methods section. After applying different algorithms available in MERCI software, such as none, Koolman–Rohm and Betts–Russell, several motifs were discovered which were found to be exclusively present in PiEs and NPiEs (Table 1; Additional file 3: Table S3). The overall coverage of a motif represents the total number of epitopes harboring that particular motif and same epitopes might also be covered by other motifs. Out of 583 PiEs present in positive dataset, the Betts–Russell algorithm, Koolman–Rohm algorithm and none algorithm could identify 256, 192 and 179 unique PiEs using 11, 10 and 10 motifs, respectively. Similarly, the above algorithms were used to identify motifs from NPiEs (Additional file 3: Table S3).

**Table 1:** Number of exclusive proinflammatory (N_*p*_) and non-proinflammatory epitopes (N_*n*_) covered by motifs identified using different algorithms of MERCI software

Algorithm for motif discovery	N_*p*_	N_*n*_
Betts–Russell	256	29
Koolman–Rohm	192	15
None	179	9

For example, Betts–Russell algorithm-based proinflammatory and non-proinflammatory motifs could identify 256 proinflammatory as well as 29 non-proinflammatory unique epitopes, respectively

The “hydrophobic hydrophobic K hydrophobic hydrophobic” and “hydrophobic aliphatic polar N” were found to be the most frequent motifs and covered 54 and 44 unique proinflammatory epitopes, respectively. Among motifs given by Koolman–Rohm algorithm, “basic A aliphatic” was the most recurring motif covering 41 unique proinflammatory epitopes. Interestingly, the 10 motifs obtained in MERCI analysis with none algorithm were same as the significant dipeptides obtained in the compositional analysis. MERCI motifs, which were discovered exclusively in proinflammatory epitopes against non-proinflammatory epitopes, showed similar conservation of residues as observed in compositional analysis, for example, the motifs having highest coverage as shown in Table 2, are abundant in hydrophobic and aliphatic residues at various positions. Similar observations were also reported earlier where MBP(85–105) peptide binds to MHC allele DRB1 * 1501 with a nonaromatic, hydrophobic anchor (L, V, or I) at position i and by a bulky hydrophobic residue (F or Y) at position i + 3 as primary anchor which may contribute to its immunodominance [26].

**Table 2:** Motifs discovered in proinflammatory epitopes along with the overall coverage for each motif

Proinflammatory MERCI motifs	Overall coverage
Hydrophobic hydrophobic K hydrophobic hydrophobic	54
Hydrophobic aliphatic polar N	48
K hydrophobic aliphatic polar	46
Hydrophobic hydrophobic K small hydrophobic	45
Aliphatic R hydrophobic hydrophobic	44
Positive tiny L	43
Polar tiny hydrophobic aromatic hydrophobic	43
K hydrophobic L	42
Hydrophobic positive tiny hydrophobic polar	42
Hydrophobic hydrophobic aliphatic polar small aliphatic	41
Hydrophobic N aromatic hydrophobic	41

### Machine learning-based classification

The preliminary analysis unveiled that the PiEs differ from NPiEs in amino acid sequence-based features, and thus, sequence-based features can be exploited for their classification into PiEs and NPiEs epitopes using machine learning-based classification. Classification models for different features were developed using 6 different machine learning techniques (SVM_light_ and RandomForest, BayesNet, NaiveBayes, IBk and J48). However, the performance of RandomForest, BayesNet, NaiveBayes, IBk and J48 models was observed to be lower as compared to SVM-based models (Additional file 4: Table S4). Therefore, SVM-based models were implemented and discussed in the manuscript. Similarly, the performance of models on alternate dataset is mentioned in Additional file 5: Table S5.

#### Amino acid composition-based models

**Fig. 3:**
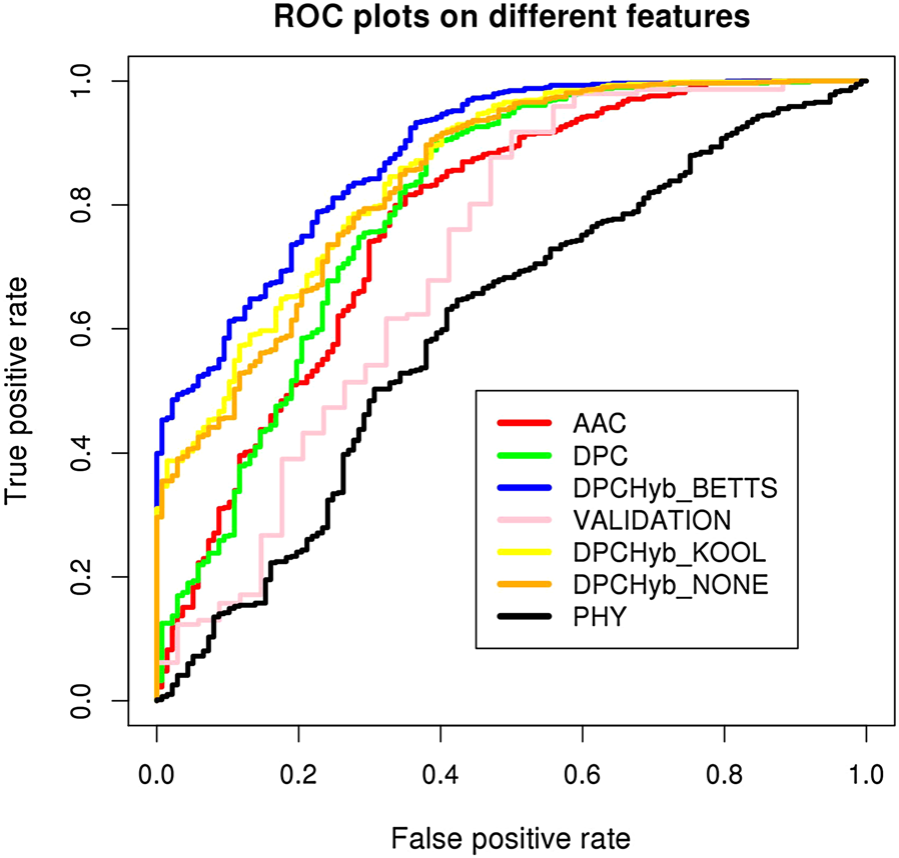
ROC plots of prediction models developed using SVMlight as machine learning technique. The DPCHyb_BETTS model (shown in *blue*) achieved highest area under curve (AUC = 0.88 as given in Table 3)

The two classes of epitopes differed in amino acid composition as mentioned in the compositional analysis (Fig. 2). Therefore, the amino acid composition was utilized to classify the two classes by developing machine learning models. After optimization of parameters, the best performing SVM-based model was selected with rbf kernel (t = 2), gamma parameter (g) = 0.005, trade off factor (c) = 80 and a cost factor (j) of 1. The model performed with an overall accuracy of 72.9 % and MCC was measured as 0.36. The threshold independent parameter area under curve (AUC) was found to be 0.77 (Table 3; Fig. 3).

**Table 3:** Performance of different classification models developed using support vector machine as machine learning technique

Feature	Thre	Sen	Spec	Acc	MCC	AUC	Parameters
Performance on training data							
AAC	0.6	73.58	70.07	72.92	0.36	0.77	t:2 g:0.005 c:80 j:1
DPC	0.4	86.11	62.04	81.53	0.45	0.8	t:2 g:0.001 c:10 j:1
PHY	0.7	91.25	24.82	78.61	0.20	0.57	t:2 g:0.001:c:50:j:4
DPCHyb_NONE	0.4	87.82	62.04	82.92	0.48	0.84	t:2 g:0.001 c:20 j:1
DPCHyb_KOOL	0.4	89.54	60.58	84.03	0.49	0.85	t:2 g:0.001 c:4 j:2
DPCHyb_BETTS	0.3	93.65	62.04	87.64	0.58	0.88	t:2 g:0.001 c:8 j:3
Performance on validation data							
DPCHyb_BETTS	0.3	91.1	50	83.33	0.43	0.71	

The hybrid model prepared using Dipeptide composition based features and MERCI displayed the best performance with an accuracy of 87.6 %. The same model showed an accuracy of 83.3 % on validation dataset

#### Dipeptide composition-based models

The models developed on AAC-based feature with vector size of 20, could not perform well both on threshold dependent as well as threshold independent parameters. Further, seeking better performance, dipeptide composition (DPC) was utilized, as input feature. The DPC-based SVM models yielded an overall accuracy of 81.5 % and MCC = 0.45 and AUC = 0.8. The optimized parameters for this model included rbf kernel (t = 2), g = 0.001, c = 10 and j = 1 (Table 3; Fig. 3).

#### Physiochemical properties-based models

While exploring different sequence-based features for better classification of PiEs from NPiEs, the physiochemical property-based models (explained in Methods) could only provide an accuracy of 79 % with MCC = 0.2, which was much lower as compared to AAC and DPC-based models (Table 3; Fig. 3) and hence, were not included in the tool.

#### Hybrid model

Among three sequence-based features mentioned above, the DPC-based model displayed the best performance. In order to further explore the possibility of better performance, the motif information was incorporated into the DPC-based models. A hybrid model of DPC and MERCI motifs were developed using the same methodology which was used to develop the DPC models and validated by tenfold cross-validation. Furthermore, three different models were developed employing three different algorithms of MERCI as described in method section. The overall accuracy of DPC-motif hybrid model with none algorithm (DPCHyb_NONE) was found to be 83 %, whereas, the DPC-motif hybrid model with Koolman–Rohm (DPCHyb_KOOL) gave an accuracy of 84 %. The DPC-motif hybrid with Betts–Russell algorithm yielded an overall accuracy of 87.6 %, MCC = 0.58 along with an AUC of 0.88 and was selected. The model was developed using rbf kernel with g = 0.001, c = 8, j = 3 (Table 3; Fig. 3).

#### Performance on validation dataset

Although, the tenfold cross validation technique is well accepted practice in machine learning methods, there are chances of over-fitting. In order to examine the possibility that the observed performance of the final model could be due to over-optimization, the model was tested on validation dataset. The DPCHyb_BETTS model displayed an accuracy of 83.3 % with MCC = 0.43 and AUC of 0.77 on validation dataset (Table 3).

The DPCHyb_BETTS model could achieve good accuracy and MCC on DPC-motif hybrid inputs. The strategy of giving weightage worked well and enhanced the performance of model in terms of accuracy, MCC and AUC. Since the prediction model performed well on unseen validation dataset, it attests that the performance of the model is not due to over optimization.

The performance of models developed on alternate dataset is mentioned in the Additional file 5: Table S5. In brief, among models developed on alternate dataset, RandomForest performed best using AAC as input feature. The accuracy of the AAC-based RandomForest model was 67.2 % with MCC = 0.34.

The DPCHyb_BETTS model (developed on main dataset) was incorporated in the webserver to allow the users to analyze and get predictions for their queries. It could serve as a computational substitute to the costly and time consuming experiments, as mentioned in case of HP(2–20) of *H. pylori*, gG2p20 of HSV-2, prion peptide PrP(106–126) and C-peptide of proinsulin. Using this web-based tool, users can sort down number of candidates responsible for proinflammatory nature of an antigen, which can further be validated by experimental studies.

### Webserver and tools

The predictive modeling yielded classification tools with good accuracy for predicting the proinflammatory property of a peptide/protein and were used to develop a webserver by incorporating prediction model along with different other analysis tools. To show the translational application of “Proinflam” web server, a highly relevant example of the C-peptide from proinsulin protein is included in different modules (Peptide prediction, Protein Scan, Epitopes mapping and Similarity Search) of the web server. The C-peptide is a byproduct of proinsulin, located at position from 57 to 87 amino acids in the proinsulin protein, and was predicted as a proinflammatory epitope from the proinsulin protein using the web server. This peptide is responsible for the proinflammatory events in kidney and vasculature leading to diabetic nephropathy and atherosclerosis, respectively [11]. This example demonstrates the application and ability of the server to predict the proinflammatory epitopes in clinically relevant real proteins.

### Peptide prediction

This module is designed for submission of single or batch of peptide/protein sequences in FASTA format with a length ranging from 4 to 30 amino acids. This tool runs the queries through the prediction pipeline with DPCHyb_BETTS model and classifies the queries into PiEs or NPiEs. The threshold option is provided to select the stringency of positive prediction.

As an additional function, virtual screening and designing option has been provided in the result table which allows the resubmission of the selected peptide. The virtual screening and designing involves substitution of amino acids at each position of peptide with the other 19 natural amino acids, which are further predicted in terms of PiEs or NPiEs and the results are displayed as a table. It allows the user to predict the proinflammatory nature of different variants of a given query.

### Protein scan

Apart from peptide prediction module, which was meant for small length peptides, this module can be used for identification of antigenic regions in a protein, which can induce proinflammatory response in a host. The provision of window length allows user to select desired length of peptide for prediction. Similar to the peptide prediction module, this module runs query through prediction pipeline and virtual screening and provides the results as a table.

### Epitope mapping

The prediction models achieved good accuracy in this study. However, user might want to investigate if there are previous reports of experimentally validated epitopes mapping to the query sequence. Therefore, ‘Epitope mapping’ module is developed to assist the user for mapping experimentally validated PiEs on the query sequence. Using this tool, the user can map the query sequence with PiEs and can also link to related assays in IEDB database.

## Similarity search

In contrast to the epitope mapping module where exact match with experimental data is carried out, the ‘Similarity Search’ module performs Smith-Waterman search of query sequence in the database of experimentally validated PiEs. The top hits are shown with alignment and links to related assays in IEDB database.

## Conclusion

The tendency of an antigen to initiate proinflammatory cascade, such as recruiting neutrophils, monocytes, and activate complement proteins, is of great importance in immunology and peptide therapeutics. Therefore, we have investigated sequence-based signatures which could be responsible for the proinflammatory nature of a peptide and developed a machine learning-based prediction tool for the prediction of proinflammatory epitopes. The computational identification of proinflammatory antigenic candidates before going for expensive and time-consuming experiments would be of great help to the scientific community. The developed computational tools are available freely for academic usage as a web server.

## Methods

### Dataset

The availability of clean experimental data is very crucial for any predictive modeling. Therefore, the Immune Epitope Database (IEDB) [27] for immune assays carried out for different peptide antigen was used to retrieve a clean dataset of 729 epitopes which were reported positive in assays in which any one of the proinflammatory cytokine [IL1α, IL1β, TNFα, IL12, IL18 and IL23] [25] was measured. Since these 729 epitopes were showing proinflammatory response in assays, these epitopes were considered as proinflammatory epitopes (PiEs) in this study. All those peptides which were assayed negative for proinflammatory cytokines were considered as non-proinflammatory epitopes (NPiEs). The total retrieved dataset contained 729 PiEs and 171 NPiEs ranging in length between 4 and 30 amino acids and was termed as ‘Main Dataset’. From the main dataset, 80 % of the data was assigned as training data and 20 % of the data was picked up randomly and kept as validation dataset (Fig. 1). The final training dataset contained 583 PiEs (positive data) and 137 NPiEs (negative data). The validation dataset contained 146 PiEs and 34 NPiEs.

Since the NPiEs (negative dataset) in main dataset was lesser in numbers as compare to PiEs (positive dataset), an additional dataset was constructed where the number of negative examples were kept equal to the number of positive examples. For this, 558 randomly picked non-T cell epitopes (NTCEs) data of 4–31 amino acid length were added to NPiEs of main dataset. The finalized alternate dataset contained 729 PiEs and 729 NPiEs (171 NPiEs + 558 NTCEs). The models were trained and tested in the same manner as done for the main dataset.

The main dataset was used for compositional and motif-based analysis, whereas, both main dataset and alternate dataset were used for the development of machine learning-based models. The models developed using the main dataset were incorporated in web server as prediction tool.

### Input features for machine learning

#### Composition-based features

##### Amino acid composition

Amino acid composition (AAC) is the percentages of each amino acid in the given length of amino acid sequence. AAC has widely been applied in different peptide and protein composition based classification method [28, 29]. Since there are 20 amino acids, each peptide/protein is represented by 20 types of compositions or a vector size of 20.

$$AAC(i) = \frac{\text{Total}\,\text{number}\,\text{of}\,\text{amino}\,\text{acid}\,\text{(i)}}{\text{Total}\,\text{number}\,\text{of}\,\text{all}\,\text{possible}\,\text{amino}\,\text{acids }} \times 100$$

where, AAC(i) is the amino acid composition of the amino acid (i) and amino acid (i) is one of the 20 amino acids.

##### Di-peptide composition

Similar to the AAC, dipeptide composition (DPC) has also been extensively applied in sequence-based classifications, particularly in the immune epitope prediction algorithms [30, 31]. DPC differs from AAC in having pair of amino acids and thus also provides information on local arrangement. The percentage of every possible pair (dipeptide) of amino acids was calculated. The following equation has been used for this calculation:

$$DPC(i) = \frac{\text{Total}\,\text{number}\,\text{of}\,\text{dipeptides}\,\text{(i)}}{{Total\,{\text{number}\,\text{of}\,\text{all}\,\text{possible}\,\text{dipeptides }}}} \times 1 0 0$$

where, DPC(i) is the dipeptide frequency of dipeptide (i) and the dipeptide (i) is one out of 400 dipeptides.

#### Physiochemical properties

In earlier studies, different physiochemical properties of amino acids have been used in several classification methods for predicting immune epitopes [24, 32] and these features are also implemented in this study for developing the prediction models. 10 different physico-chemical properties were computed, namely amphipathicity, hydrophobicity, pI value, bulky side chain, hydrophilicity, net-hydrogen, steric hindrance, charge, hydropathy, molecular weight [32, 33].

#### Motif-based features

Identification of functional motifs in amino acid sequences has widely been exploited in functional annotation of protein/peptide sequences. Several authors have discovered immunologically relevant motifs in immunoinformatics studies [19, 34]. In this analysis, motifs specific to PiEs were identified using MERCI software (http://dtai.cs.kuleuven.be/ml/systems/merci) [35]. MERCI software is a tool to identify exclusive motifs present in positive data by comparing it with negative data. The exclusive motif discovery using MERCI was carried out using two steps. In the first step, PiEs were taken as positive and NPiEs were taken as negative data, which yielded exclusive motifs present in PiEs. In the second step, in order to get the exclusive motifs present in NPiEs, the datasets were reversed, i.e. NPiEs as positive data and PiEs as negative data.

While discovering the motifs with MERCI software, three algorithms were adopted: (a) none (b) Koolman–Rohm, and (c) Betts–Russell. The length of motif was set as maximum of 9 amino acids because the size of binding core in both MHC I and II is 9 amino acids [36, 37]. The gap length was set to default (1).

#### Hybrid feature

The hybrid of compositional features and motif-based feature has already been used in various prediction tools by different authors [19, 20]. In this study, a hybrid of DPC and MERCI-based motifs was used to improve the classification performance. As described above, two sets of motifs were identified as exclusively found in PiEs and NPiEs, respectively. In order to make a hybrid feature, the presence of proinflammatory and non-proinflammatory motifs was searched in the peptide. If the peptide is positive for proinflammatory motif, the weight of +1 was assigned to the DPC based SVM score. Similarly, if the peptide is positive for non-proinflammatory motif, a weight of −1 was assigned to the SVM score.

### Machine-learning-based prediction models

#### Support vector machine

In this study, support vector machine is used as the machine learning algorithm implemented using SVMlight package available at http://svmlight.joachims.org/. SVM-based models are trained with a learning algorithm where it draws an optimal hyperplane in a multi-dimensional feature space that creating a boundary dividing the datasets in two classes. Among different machine learning techniques, SVM performs well because it is effective in controlling the classifier’s capacity and associated potential for overfitting [38]. In particular, SVM has been hugely implemented in various immune epitopes prediction tools [31, 39], protein structure prediction [40] and genomic data [41] because of its ability to handle noise and large dataset [42].

#### RandomForest

In this study, RandomForest (RF) has been implemented using randomForest package in R (http://cran.r-project.org//). RF has been widely used for the binary as well as multiclass classification using nucleotide or amino acid compositions as feature inputs [43]. RF classification model, at optimized parameters with lowest out-of-bag (OOB) error, was selected for the classification purpose. The overall performance of the selected model was evaluated in terms of sensitivity, specificity, accuracy and MCC from the confusion matrix.

#### WEKA-based techniques

In addition to the SVM and RF, BayesNet, NaiveBayes, IBk (kNN) and J48 machine learning algorithms were also evaluated through WEKA package. These techniques have already been implemented in immune epitope prediction studies in earlier studies [31, 44]. Similar to SVM and RF, the performances were evaluated using sensitivity, specificity, accuracy, MCC and AUC.

#### Evaluating performance of models

Evaluation and comparison of learning methods is essential part of predictive modeling. Cross-validation technique is among most practiced techniques which involve dividing the data into two segments; the first part is used to train the model and the other holdout or test data is used to test the model. Tenfold cross validation technique has been adopted where at a given instance, nine segments were used in training the model and the rest one segment was used to test the model. The process is repeated ten times such that each segment can be tested. The performance of model was calculated by including results from all the ten predictions taken together. The performance of models can be measured by both threshold dependent as well as threshold independent parameters. For threshold independent parameter, AUC was measured which was calculated by PERF software. For threshold dependent parameters, the parameters like sensitivity (Sen), specificity (Spec), accuracy (Acc) and Matthews’s correlation coefficient (MCC) were calculated. The following equations were used for the calculation of these parameters:

$$\begin{aligned} & Accuracy = \frac{TP + TN}{TP + FN + FP + TN} \\ & Sensitivity = \frac{TP}{TP + FN} \\ & Specificity = \frac{TN}{TN + FP} \\ & MCC = \frac{{\left( {TP \times TN} \right) - \left( {FP \times FN} \right)}}{{\sqrt {\left( {TP + FP} \right)\left( {TP + FN} \right)\left( {TN + FP} \right)\left( {TN + FN} \right)} }} \\ \end{aligned}$$

where, TP = True Positive, FP = False Positive, FN = False Negative, TN = True Negative.

## Additional files

10.1186/s12967-016-0928-3 Amino acid composition distribution between proinflammatory and non-proinflammatory data.
10.1186/s12967-016-0928-3 Dipeptide composition distribution between proinflammatory and non proinflammatory data.
10.1186/s12967-016-0928-3 MERCI motifs extracted form proinflammatory and non-proinflammatory data.
10.1186/s12967-016-0928-3 Performance of models developed on main dataset, with RandomForest, BayesNet, NaiveBayes, IBk and J48.
10.1186/s12967-016-0928-3. Performance of different machine learning models developed on alternate dataset.

## Abbreviations

AAC: amino acid composition
Acc: accuracy
AUC: area under curve
DPC: dipeptide composition
IEDB: immune epitope database
MCC: Matthews correlation coefficient
MHC: major histocompatibility complex
NPiEs: non-proinflammatory epitopes
NTCEs: non T Cell epitopes
OOB: out-of-bag
PHY: physiochemical properties
PiEs: proinflammatory epitopes
RF: RandomForest
Sen: sensitivity
Spec: specificity
SVM: support vector machine
